# Fanconi anemia pathway as a prospective target for cancer intervention

**DOI:** 10.1186/s13578-020-00401-7

**Published:** 2020-03-16

**Authors:** Wenjun Liu, Anna Palovcak, Fang Li, Alyan Zafar, Fenghua Yuan, Yanbin Zhang

**Affiliations:** 1 Department of Biochemistry and Molecular Biology, University of Miami Miller School of Medicine, Gautier Building Room 311, 1011 NW 15th Street, Miami, FL 33136 USA; 2 Sylvester Comprehensive Cancer Center, University of Miami Miller School of Medicine, Miami, FL 33136 USA

**Keywords:** Fanconi anemia, DNA repair, Tumorigenesis, Cancer intervention

## Abstract

Fanconi anemia (FA) is a recessive genetic disorder caused by biallelic mutations in at least one of 22 FA genes. Beyond its pathological presentation of bone marrow failure and congenital abnormalities, FA is associated with chromosomal abnormality and genomic instability, and thus represents a genetic vulnerability for cancer predisposition. The cancer relevance of the FA pathway is further established with the pervasive occurrence of FA gene alterations in somatic cancers and observations of FA pathway activation-associated chemotherapy resistance. In this article we describe the role of the FA pathway in canonical interstrand crosslink (ICL) repair and possible contributions of FA gene alterations to cancer development. We also discuss the perspectives and potential of targeting the FA pathway for cancer intervention.

## Introduction

Normal cells harbor a delicate system of DNA damage sensing and repair to overcome a variety of DNA lesions that otherwise elicit genome instability and cellular toxicity if not repaired properly [1, 2]. A hallmark of many cancers is genome instability which results from dysregulation of DNA damage sensing and repair [3]. Clonal selection of advantageous mutation carriers with compromised cell cycle checkpoint, constitutive pro-proliferative signaling, or dysfunctional cell death constitutes the onset of somatic carcinogenesis [4]. Meanwhile, elevated or rewired DNA repair networks function as caretakers to handle excessive DNA damage and replicative stress resulting from rapid cancer cell proliferation. This reprogramming is frequently found to be associated with resistance to common chemotherapies [5]. The Fanconi anemia (FA) pathway is known for its role in DNA interstrand crosslink (ICL) repair [6]. In addition to its pathological relevance to the genetic disorder of Fanconi anemia, the FA pathway has been overwhelmingly positioned in the context of cancer [7, 8], suggesting that targeting the FA pathway is a prospective avenue for cancer intervention.

## The FA pathway and interstrand crosslink repair

ICLs are a class of DNA lesions that can be introduced both endogenously and exogenously. Aldehydes, which are produced by many metabolic processes such as lipid peroxidation, histone demethylation, and alcohol metabolism, cause the formation of ICLs [9–12]. Common chemotherapeutic agents such as mitomycin C and platinum are DNA crosslinkers that introduce both intrastrand crosslinks and ICLs. While intrastrand crosslinks are readily repaired by the nucleotide excision repair (NER) pathway [13], ICLs represent a highly cytotoxic lesion that is primarily repaired by the FA pathway [14]. Fanconi anemia is a rare genetic disorder caused by biallelic mutations in one of the 22 known FANC genes [15–21]. Affected patients have deficient ICL repair. Clinical diagnosis of FA can be performed through observation of elevated chromosomal rearrangements (predominately radial chromatids) within patient derived cells after treatment with an ICL-inducing agent such as Diepoxybutane [22, 23]. The 22 FANC proteins along with many FA associated factors work together to recognize ICL damage, activate the pathway by FANCI-FANCD2 (ID2) monoubiquitination, and initiate downstream double-strand break (DSB) repair.

### ICL recognition and the FA core complex

**Fig. 1:**
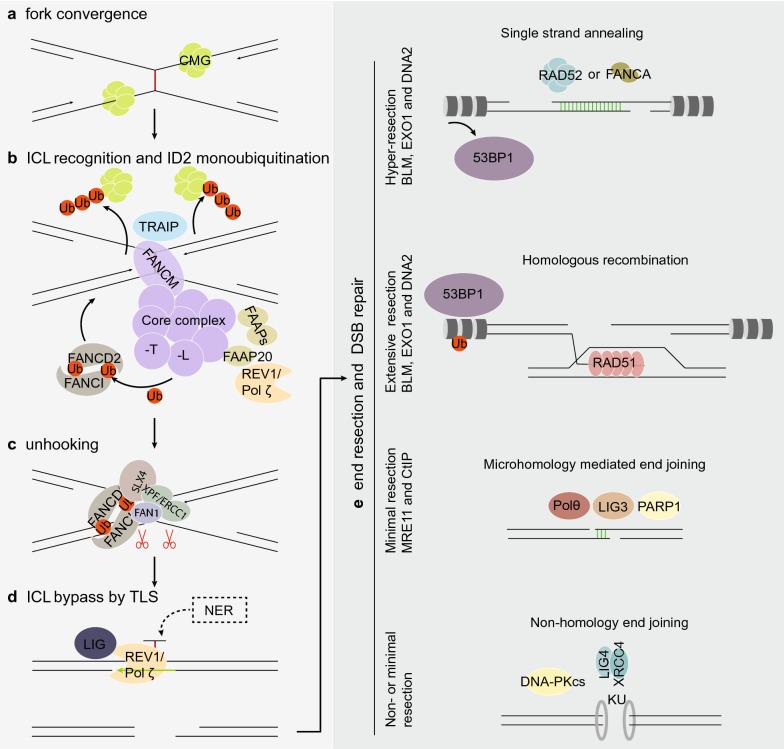
The Fanconi anemia pathway of DNA repair. **a** Repair of ICL in replicative phase starts with convergence of two replication forks surrounding the ICL site. **b** FANCM/FAAP24 complex recognizes the X shaped DNA structure and recruits other members of the FA core complex and FAAPs. Monoubiquitination of the ID2 complex represents the step of activation of the FA pathway. **c** ID2 recruits FAN1 and structure specific nucleases for incision surrounding the ICL sites to unhook the damage. **d** Translesion synthesis polymerases REV1/pol ζ bypass the unhooked ICL damage. The unhooked ICL remnant will be subsequently repaired by NER. **e** Double strand break intermediates can be repaired via four sub-pathways of DNA DSB repair depending on the result of end resection

ICLs that occur outside of S phase are sensed and repaired by the NER pathway [14]. The FA pathway-mediated ICL repair occurs primarily in S phase and starts with the formation of an X shaped DNA structure that occurs upon convergence of two head-on replication forks surrounding the ICL site [24, 25] (Fig. 1a). The CMG (Cdc45-MCM-GINS) helicase complex is ubiquitinated by the E3 ubiquitin ligase TRAIP [26]. Short ubiquitin chains recruit NEIL3 glycosylase for incision-independent unhooking mechanism of ICL resolution. Long ubiquitin chains promote CMG unloading from the chromatin in a p97 dependent manner [26, 27] which allows further approach of the two replication forks toward the ICL (Fig. 1b), and commitment to Fanconi anemia pathway-mediated ICL repair. It has been demonstrated that the FANCM/FAAP24 complex recognizes the ICL lesion and initiates recruitment of other components of the FA core complex [28] (Fig. 1b). The FA core complex assembles through several sub-complexes: FANCA-FANCG-FAAP20, FANCE-FANCF-FANCC, FANCB-FANCL-FAAP100 [29–33], and other FAAPs. A most recent structural study described an 8-FA-protein core complex that assembled surrounding the scaffold comprised of two central heterodimers of FANCB and FAAP100. FANCB and FAAP100 adopt similar structure despite limited sequence homology [30]. Two RING finger FANCL subunits flank the FANCB-FAAP100 scaffold in different conformations suggesting functional asymmetry. Selective incorporation of the E2 ubiquitin-conjugating enzyme UBE2T (FANCT) into the core complex by the E3 ubiquitin ligase FANCL determines substrate specificity and modification type [34]. Mutations or loss of any core complex components lead to diminished monoubiquitination activity and thus inefficient activation of the FA pathway. As the activation signal in the FA pathway, FANCD2 and FANCI (ID2) are monoubiquitinated on Lysine-561 and -523 respectively [35–38] by FANCL and UBE2T. The most recent structure-based study suggests that the monoubiquitination of FANCD2 during DNA repair stabilizes a closed clamp conformation of the FANCI-FANCD2 complex [39]. Under non-damaging conditions, accessibility to these residues are buried in the complex. Damaging conditions allow guidance from ATR-CHK1-mediated FANCI phosphorylation that promotes ID2 dissociation and interface exposure [40, 41] (Fig. 1b). Meanwhile, modification of FANCD2 allows a functional switch where a cluster of phosphorylation sites between residues 882 and 898 are substrates of casein kinase 2 (CK2) [42]. Upon completion of ICL repair FANCD2 is deubiquitinated by chromatin bound USP1-UAF1-RAD51AP1 complex and released from the initial damage site for the next round of repair events [43–45].

### Unhooking and translesion synthesis (TLS)

FANCD2 binds to H4K20me2 via its histone-binding domain (HBD) and an embedded methyl-lysine-binding domain (MBD). A HBD/MBD mutant of FANCD2 that can be efficiently monoubiquitinated demonstrates impaired chromatin binding and foci formation, suggesting that monoubiquitination precedes ICL recruitment of FANCD2 [46]. However, arguing against this time course are studies that showed the ID2 complex is recruited to ICLs before the occurrence of FANCD2 monoubiquitination [41]. Nevertheless, ubiquitinated ID2 complex is required for the recruitment of structure specific endonucleases (SSEs) and translesion synthesis (TLS) polymerases for downstream repair [47]. Removal of the ICL starts with nucleolytic cleavage at stalled forks to incise the ICL on one parental strand, a process known as unhooking (Fig. 1c). ERCC1/XPF (FANCQ), MUS81/EME1, and FAN1 have been implicated as necessary for ICL incision [2, 14, 48–60]. SLX4 (FANCP) is the master scaffold and regulator of ERCC1/XPF, MUS81/EME1/2, and SLX1 nucleases for ICL processing [7]. SLX4 enters the ICL site through its N-terminal ubiquitin-binding zinc finger (UBZ) domain [61] and further recruits and activates the ERCC1/XPF (FANCQ) endonuclease activity [62]. The mechanism for selective recruitment of downstream nucleases by SLX4 remains elusive. Nevertheless, a recent study suggests that SLX4IP, a constitutive member of the SLX4 complex promotes ERCC1/XPF incorporation [63].

Following unhooking, the ICL remnant on one parental strand becomes a roadblock for replicative polymerases. Translesion synthesis polymerases are thus recruited to the ICL site by the FA core complex [64] for damage bypass. A dCMP transferase Rev1 first inserts a dCMP opposite the unhooked ICL and extension of the DNA synthesis is carried out by pol ζ [64, 65]. Damage-induced mutations are introduced surrounding the ICL position with a mutation frequency of ~ 1% [64]. NER mediated removal of the ICL remnant on the parental strand completes the repair (Fig. 1d). The other DNA duplex with a DSB is ultimately repaired by multiple DSB repair sub-pathways.

### DSB repair through HR and other sub-pathways

DNA DSBs can be repaired through four coexisting sub-pathways, namely homologous recombination (HR), single strand annealing (SSA), microhomology-mediated end joining (MMEJ), and non-homologous end joining (NHEJ) that require differentially resected DSB ends and different levels of homology [66, 67] (Fig. 1e). Ku proteins readily bind minimally- or non- resected DSBs based on their abundance and low *K*_d_ for DNA ends [68–70], and recruit DNA-dependent protein kinase (DNA-PKCs) and DNA ligase-4 (LIG4) to carry out non-homologous end joining (reviewed in [24]). Alternatively, a DSB undergoes progressive end resection. MRE11 and CtIP nucleases generate minimally resected DSB ends with 3′ ssDNA. Helicases and exonucleases BLM, EXO1 and DNA2 are brought in to produce extensively resected DSB ends [71]. Further resection is prevented by the presence of chromatin bound 53BP1 and 53BP1-recruited assembly of RIF1, REV7, PTIP, and Artemis [72]. Hyper-resection occurs when 53BP1 is unloaded from chromatin flanking the DSB. These differentially processed DSB ends are preferred substrates for MMEJ [73], HR, and SSA [74] respectively (Fig. 1e).

HR and NHEJ have been known as the two sub-pathways of DSB repair that are more relevant for FA pathway-mediated ICL repair. Among the 22 FA complementation groups a large number are well-established HR factors. HR is known as the preferential pathway over error-prone end joining for the repair of DSB intermediates that occur during ICL repair (reviewed in [20, 75]). This opinion is supported by the readily available HR compatible template (the repaired DNA duplex) that occurs at ICL sites, and many other experimental observations. Monoubiquitinated FANCD2 has been shown to interact with CtIP nuclease [76], one of the early exonucleases needed to initiate end resection of DSBs and disable NHEJ. FA pathway deficiency not only causes reduction in HR efficiency in the DR-GFP assay [77, 78], but also leads to increased deleterious repair through NHEJ, which is largely responsible for the translocations and abnormal chromatin structures observed in FA patient cells [75]. Accordingly, ICL sensitivity can be rescued by inhibition of many NHEJ factors in many FA deficiency models including C. elegans, chicken DT40 cells, mouse embryonic fibroblasts and human cells where FA components were knocked down, knocked out, or mutated [79, 80]. Besides these two subpathways, SSA may also participate in ICL and DSB repair via the newly identified strand annealing activity of FANCA [66, 81, 82].

## The relevance of the FA pathway to cancer

Blood and bone marrow stem cell transplants are the most effective treatment for various Fanconi anemia cases and confer significant improvement for quality of life and lifespan of FA patients. A significant cause for the death of FA patients has shifted to cancer development associated with failure of the FA repair pathway. A most recent survey of 111 FA patients indicates a cancer frequency of 30%. Intriguingly FA-A patients develop cancer at the age of 18.5 (mean), significantly older than 5.2 (mean) for the other complementation groups [83]. Myeloid leukemias, liver tumors, head and neck carcinomas, and gynecologic malignancies are the most profound predisposing cancers among FA patients [7, 84]. Sequencing studies and FISH analysis have shown that amplifications of certain oncogenes due to chromosomal instability are at least partially responsible for blood cancers in FA patients [85].

Over the last two decades, many Fanconi anemia mouse models have been employed to study the pathology, and explore the clinical managements of FA (see reviews [86, 87] with systematic survey of early mice studies). While cells derived from knockout mice recapitulate the phenotypes of FA patient cells in general, these mice also partially reproduce the pathological characteristics of FA patients especially for the hematological abnormality and cancer occurrence, albeit with lower onset incidence and dissimilar cancer types. This allows valuable evaluations of chemo- and radio-therapy efficacy for cancers and genotoxicity alleviation in FA patients as FA carriers are hypersensitive to DNA lesions.

Beyond the disease of Fanconi anemia, more intriguingly somatic alteration of FA genes has been widely characterized in cancer tissues by large scale sequencing. The role of FA genes in cancer development is discussed below.

### The FA pathway protects cells from R-loop accumulation and genome instability

Genome instability is a hallmark of cancer [88]. The FA pathway is a major player for the maintenance of genome stability through DNA damage repair, replication fork stabilization, and oxidative and mitotic stress alleviation (see our previous comprehensive review [75] and [7]). A R-loop is a 3-stranded DNA:RNA hybrid structure produced co-transcriptionally. R-loop formation represents a cellular process for gene expression regulation as well as a major source of genome instability [89–92]. R-loop accumulation largely results in and from collisions that occur between replication forks and the transcriptional machinery in a head-on orientation [93]. One emerging function of the FA pathway is to protect cells from R loop accumulation and its associated genome instability. Both human and murine FA deficient cells (FA-A, FA-D2, FA-M) exhibit elevated levels of R-loops and genome instability [92, 94]. Monoubiquitination of FANCI-FANCD2 complex can be enhanced upon their binding of both RNA and R-loop substrates in vitro [95]. It has been shown that RNA processing factors such as hnRNP-U and DDX47 are recruited by FANCD2 for R-loop resolution [96, 97]. Besides RNaseH1, a predominant factor for efficient R-loop removal, an alternative resolution of R-loop is through the translocase activity of FANCM, as FANCM catalyzes displacement of RNA from the R-loop structure [94]. These data suggest that many FA components or the whole FA pathway participate in R-loop suppression.

### Association of FA alterations with somatic cancer

**Fig. 2:**
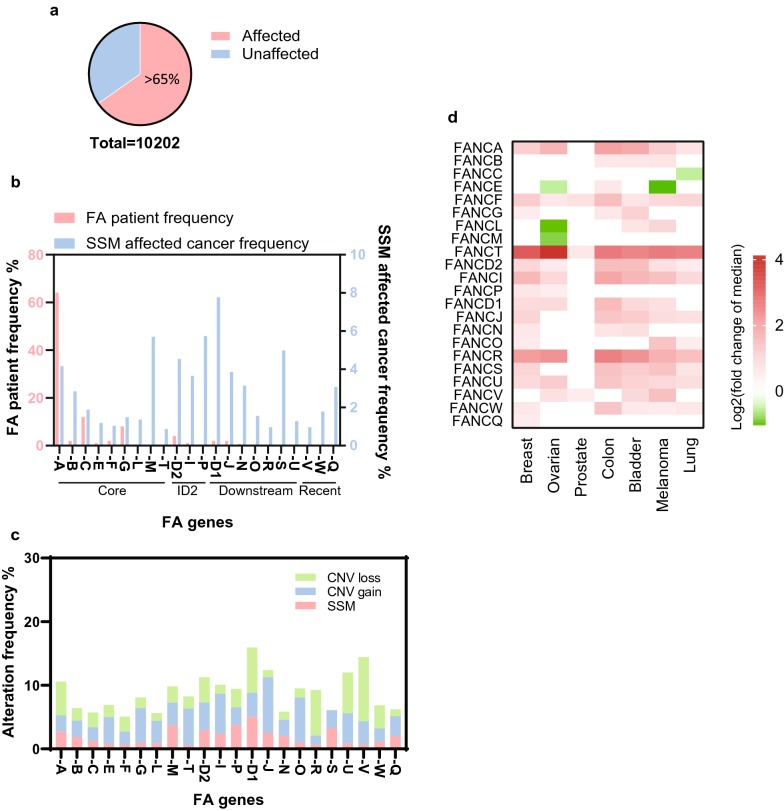
Alterations in FA genes are common in cancers. **a** Among the surveyed 10,202 tumors (no tissue origin filter) in the database of TCGA over 65% carry FA gene alterations (Affected). **b** The patient frequency of FA and frequency of FA SSM-affected cancers (total 6653 FA affected cases) are superimposed to reveal a significantly different distribution across the 22 FA genes. **c** Stacked column presentation of the frequency of copy number loss, copy number gain and SSM among FA affected cancer cases. **d** Differential gene expression with p value under 0.05 and log2 (fold change) > 0.5 of every FA gene within a panel of 7 cancer types were plotted in a heatmap fashion

Beyond germline mutations in FA patients, sporadic alterations of FA genes are frequently found in many cancers. A brief survey of cases in the NIH GDC portal reveals that over 65% of the 10,202 listed cancers have at least one alteration of one of the FA genes (mutation, gain or loss) (Fig. 2a). Alterations of FANCA, FANCC and FANCG are the most predominately observed FA mutations and account for over 80% of Fanconi anemia patient cases. Simple somatic mutation (SSM) frequency of FA genes in the cancer population shows a rather even distribution among FA genes, with modest elevation in the ID2 complex, FANCA and FANCM of the core complex, and some downstream HR components (Fig. 2b). This discrepancy in mutation distribution in cancer and Fanconi anemia suggests that molecular actions of FA genes during FA development and cancer development are different.

### Two-tier contribution of the FA pathway to cancer

In addition to mutations, copy number changes (both gain and loss) of FA genes are also commonly present in cancer (Fig. 2c). Meanwhile, a survey of differential gene expression (tumor vs normal) of individual FA genes by using tumors and tissues from the TCGA and GTEx database, respectively, clearly indicates overexpression of most FA genes across a variety of cancer types (Fig. 2d). Consistent with the prevalent upregulation of almost all FA genes in cancer, is that FA gene expression receives coordinated regulation through the Rb/E2F pathway [98]. The coexistence of both gain (copy number increase and RNA expression increase) and loss (copy number loss and mutations) of function alterations for almost every FA gene with no obvious preference suggests that the distribution of FA genes to cancer is two-tier. On one hand, deficient FA pathway and rerouted damage repair pathways cause genome instability and “mutator phenotype” resulting in accumulation of mutations, deletions and translocations that constitute the starting pool for tumorous clonal selection. On the other hand, elevation of FA gene expression and thus DNA damage repair capacity is beneficial for mitigating excess DNA lesions and chromosomal abnormalities that accompany fast proliferation of cancer cells [5]. This divergence is reconciled under a model of biphasic requirement of the FA pathway throughout the initiation and progression of cancer, reminiscent of stage-specific alteration of FANCF expression by promoter methylation in ovarian cancer [99].

### Transcriptional regulation functions of FA proteins

In addition to ICL repair, a handful of studies also imply that the FA pathway is involved in transcriptional regulation that may contribute to cancer development. The FA core complex interacts with transcriptional repressor Hairy Enhancer of Split 1 (HES1) [100], binds to the HES1 promoter, and regulates HES1 responsive genes directly and indirectly [101]. FANCC regulates nuclear translocation of β-catenin and works as a transcriptional repressor of β-catenin downstream gene DKK1 [102]. Besides these case studies, An RNA-seq study revealed a collection of genes with altered expression among which a large portion are implicated in oncogenic processes [103]. Further studies are needed to explore the potential transcriptional regulation role of FA proteins especially in cancer contexts where FA gene expression is found to be high.

## The FA pathway as a prospective target for cancer intervention

### Association of the FA pathway with drug resistance

One way for cancer cells to become refractory to DNA damage-inducing chemotherapy is through acquisition of higher DNA damage repair capacity. Platinum based compounds, such as cisplatin, have been widely used to treat various cancers [104]. However, their potency is often challenged by acquired resistance [104–106]. Elevation of FA gene expression is pervasive in cancers (Fig. 2a) and is frequently found to be associated with chemo-resistance. A subset of ovarian cancer cell lines with FANCF methylation are hyper-sensitive to cisplatin while FANCF complementation in these cells restores resistance [99]. Similar resistance has also been found in the subset of cell lines harboring aberrant demethylation of the FANCF gene [99]. An A549 NSCLC cell derived cisplatin resistant cell line A549/DR exhibits upregulation of multiple FA genes and elevated FANCD2 monoubiquitination compared to its parental and other NSCLC lines [107]. Knock down of FA genes successfully re-sensitizes A549/DR cells to cisplatin treatments. Meanwhile, enhanced FA pathway activation has also been shown to be associated with resistance to melphalan in multiple myeloma [108] and pancreatic cancers [109]. In addition to DNA crosslinkers, the FA pathway also confers resistance to DNA alkylating agents in glioma [110]. Alongside FA-mediated drug resistance are observations that FANCA and FANCT/UBE2T correlate with poor prognosis and survival of cancer patients [111, 112]. These pre-clinical data highlight the need for development of FA targeted drugs in circumstances where chemotherapy resistance emerges as a result of elevated FA pathway expression and function.

### Exploring the FA pathway for synthetic lethality

When one cellular function is dependent on multiple pathways in parallel, activation of either pathway can be sufficient for the fulfillment of this function, and thus cell viability. Disruption of one pathway frequently enhances cell dependency on compensatory pathways. Inactivation of isolated DNA repair pathways and its associated genome instability is a common hallmark for cancer and crucial for cancer initiation and promotion [113, 114]. High levels of association of cancer types to deficiency in DNA repair pathways are widely known: FA/HR pathway (BRCA1, BRCA2, PALB2) for breast, ovarian and prostate cancer; mismatch and base excision repair (MSH2/6, MUTYH) for colorectal cancer; DNA damage response (DDR) genes (ATM) for leukemia and etc. Inactivation of these pathways provides a prominent advantage to use the synthetic lethal approach for treatments. The most successful practice of synthetic lethality is using PARP (poly (ADP-ribose) polymerase) inhibitors for BRCA deficiency-carrying cancers [115]. Abrogation of SSB repair by PARP targeting causes overwhelming DSB accumulation during replication that cannot be effectively resolved through the homologous recombination pathway and eventually leads to cytotoxic DNA abnormalities as a product of non-faithful end joining [116]. This approach confers lower systematic toxicity than traditional chemotherapies as normal cells survive the PARP inhibition when they are out of cell cycle and maintain functional homologous recombination capability. In addition to the BRCA deficient gynecologic cancers, PARP synthetic lethality has shown effectiveness with PTEN mutated cancers [117, 118], although the mechanism remains to be studied [119].

Multiple genes of the FA pathway have been highly ranked as genes of interest with synthetic lethality potential in a computational study [120]. siRNA screening reveals many genes, TREX2, PARP1, PLK1, UBE2B, ATM and more are synthetic lethal with FA deficiency [121]. Experimental models of both human fibroblasts and murine embryonic fibroblasts with FA pathway deficiency are hypersensitive to ATM inhibition [121]. Moreover formaldehyde catabolism has been shown as a prospective target for FA deficient cells to achieve synthetic lethality [122]. While the FA pathway synthetic lethality relationships are still under evaluation, these studies do encourage an inclusion of FA genes as part of tumor mutation screening for possible treatment strategy based on synthetic lethality. Vice versa, suppression of the FA pathway by FA targeting intervention is plausible for killing of cancer cells with deficiencies in synthetic lethal partner pathways of the FA pathway.

### Targeting the FA pathway with FA specific inhibitors

**Fig. 3:**
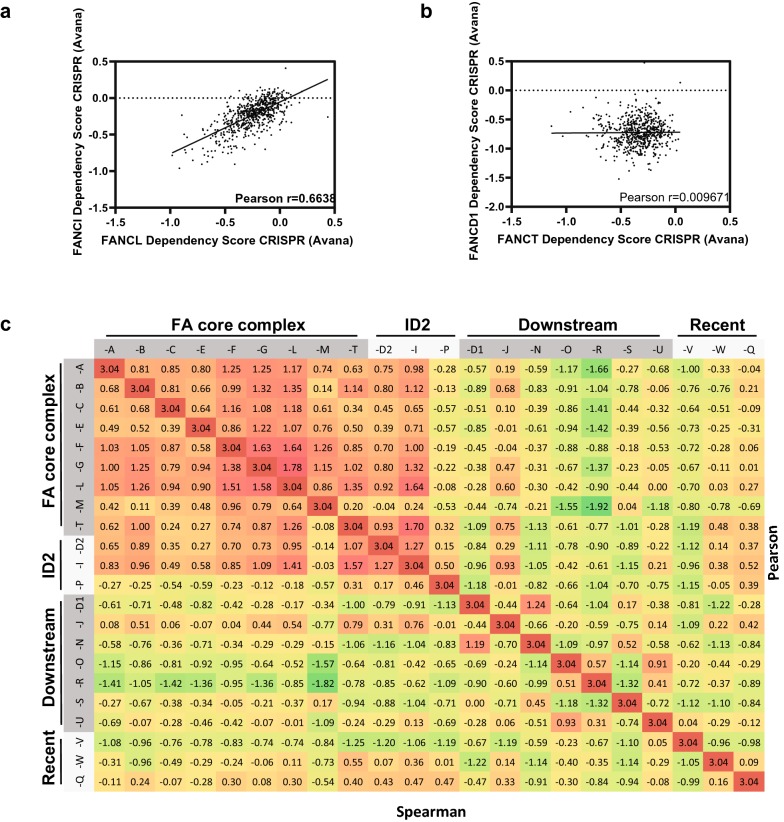
Clustering dependency analysis of FA core complex and ID2 complex components in multiple cancer lines by CRISPR knockout. Coefficient of paired dependency scores is evaluated systematically within 22 FA complementation groups. Wihle high coefficient suggests functional equality, low coefficient implies functional divergence. **a** FANCL and FANCI dependencies across over 600 cancer lines are strongly correlated. **b** FANCT and FANCD1 dependencies are poorly correlated. **c** Z scores of paired correlation efficiency scores (either Pearson or Spearman) are plotted in a diagonal table with color highlight. A clustering of FA core and ID2 complex components suggests similar cellular consequence when either individual gene is inhibited

The FA pathway is composed of 22 FA proteins and many FAAPs and operates in a progressive multistep manner. Will targeting any member impact cancer survival? To answer this question we set out to survey dependency scores (experimental measurement of genetic vulnerabilities by using CRISPR) of every FA genes within a panel of over 600 cancer cell lines of various tissue origins by using the depmap portal (https://depmap.org/portal/, [123]). While all the 22 FA genes alone exhibit only low to modest cancer cell dependency in general, considerable pairs of FA genes display highly correlated (Pearson r > 0.5) dependency across cell lines, such as the pair of FANCl and FANCL (Pearson r = 0.664, Fig. 3a) suggesting similar consequence when either one is inhibited. Nevertheless, low or non-correlation is also observed for many FA gene pairs, such as FANCT and FANCD1 (Pearson r = 0.010, Fig. 3b). When the correlation coefficients are plotted as Z scores and color highlighted, a cluster of FA genes emerges with mutually correlated dependency that comprise FA core complex and ID2 genes (Fig. 3c). This suggests comparable, if not the same outcomes can be achieved when either gene in the cluster is inhibited. Targeting members in this cluster is preferable if FA-specific effects are demanded. Divergent dependency patterns of the downstream and recently discovered FA genes reflects the complexity of these genes’ role in FA nonspecific pathways. Targeting these members might be beneficial when additional or wide spectral outcomes are favored.

While both chemotherapy re-sensitization and synthetic lethality will benefit from FA targeting inhibitors, no widely accepted FA specific therapeutic compound exists. Proteasome inhibitors are known to inhibit damage-induced FANCD2 foci formation, albeit through an unclear mechanism [124]. A few natural compounds including curcumin and its derivatives have been identified as FA pathway inhibitors through cell-based [105, 125] and Xenopus egg extract-based screening [126]. Meanwhile HSP90 facilitates FA pathway function as FANCA is a client of HSP90 and needs HSP90 interaction for stability [127]. HSP90i and withaferin A abrogate DNA damage-induced FA activation [128]. These inhibitors are capable of chemotherapy sensitization to crosslink-like damage [105, 124, 126]. However, only a handful of FA protein targeting compounds have been described (summarized in Table 1). Discovery of novel FA specific inhibitors with improved binding kinetics and FA pathway disruption is in demand. According to Fig. 3, rational inhibition of core complex and ID2 proteins are likely to sensitize cells to FA-specific impacts whereas the intervention at downstream members will be possibly beneficial for a variety of other pathways.

**Table 1:** A summary of reported FA proteininhibitors and available structures or biochemical activities to facilitate drug development

FA genes	Protein structures	Molecular activities	Inhibitors	References	
FANCA	6LHS	DNA binding, strand annealing and exchange	HSP90i including withaferin A	[66, 81, 128, 132]	
FANCF	2IQC		Natural compounds from Wrightia religiosa	[133, 134]	
FANCL	3ZQS, 4CCG	E3 ligase	CU1, CU2	[34, 131, 135]	
FANCM	4BXO, 4DAY, 4DRB, 4E45, 4M6W	DNA binding	MM2 peptide	[136–141]	
FANCT/UBE2T	1YH2, 4CCG, 5NGZ, 5OJJ	E2	A few leads by fragment screening	[34, 142–144]	
FANCD2	3S4W	DNA binding		[145]	
FANCI	3S51	DNA binding	Undisclosed	[146, 147]	
FANCP/SLX4	4M7C, 4UYI, 4ZOU	DNA binding		[148]	
FANCD1/BRCA2	1N0W, 3EU7	DNA binding	Antisense oligonucleotide (ASO)	[149–151]	
FANCJ/BRIP1	1T15, 1T29, 3AL3	Helicase		[152–154]	
FANCN/PALB2	2W18, 3EU7	DNA binding		[150]	
FANCO/RAD51C		DNA binding			
FANCR/RAD51	1B22, 1N0W, 5H1B, 5H1C, 5JZC, 5NP7, 5NWL	Strand exchange	RI-1, RI-2, B02, CYT01A	[149, 155–161]	
FANCS/BRCA1	1JM7, 1JNX, 1N5O, 1OQA, 1T15, 1T29, 1T2U, 1T2V, 1Y98, 2ING, 3COJ, 3K0H, 3K0K, 3K15, 3K16, 3PXA, 3PXB, 3PXC, 3PXD, 3PXE, 4IFI, 4IGK, 4JLU, 4OFB, 4U4A, 4Y18, 4Y2G, 6G2I	DNA binding		[152, 153, 162–174]	
FANCV/REV7	3ABD, 3ABE, 3VU7, 4EXT, 4GK0, 4GK5, 5XPT, 5XPU, 6BC8, 6BCD, 6BI7	DNA binding	REV7 specific compounds	[175–180]	
FANCW/RFWD3	6CVZ	E3			
FANCQ/XPF	1Z00, 2A1J, 2AQ0, 2KN7, 2MUT	DNA incision		[181–185]	

Multiple approaches can be employed for the development of FA specific inhibitors. Among the 22 FA genes, 13 have partially resolved structural data (alone, or complexed with other complementation groups, summarized in Table 1) that could facilitate rational design of small molecular compound leads. For instance, structural perturbation surrounding ERCC1 Phe293 is sufficient for its disruption of its interaction with XPF according to mutagenesis data [129, 130] and available structures (Table 1). Moreover, complex and functional unit formation in vitro and in vivo can be utilized for screening of interaction-disrupting compounds given proper design of compatible high throughput assays, such as fluorescence activation or FRET. In addition, a few FA proteins possess particular biochemical activities including strand annealing and strand exchange activity of FANCA, ubiquitin conjugation or ligation of FANCL, FANCT and FANCW, helicase activity of FANCJ, and even DNA binding activity of multiple FA proteins (summarized in Table 1) whose proper reconstitution in vitro can expedite drug discovery eminently. For instance, in vitro reconstitution of E1, E2/FANCT, E3/FANCL cascades has been successful in finding two hits that reduce FANCD2 foci formation and synergize with carboplatin for cancer cell killing [131].

## Conclusion

Cancer predisposition is a common genetic vulnerability beyond anemia and bone marrow failure in FA patients with inherent biallelic alterations in FA genes. Observation and association of loss of function of FA genes by means of genetic alteration and repressed transcription in many somatic cancers implies that FA inactivation may represent a more pervasive avenue for combating the onset of cancer development. Many preclinical studies demonstrated that treatment of this subtype of cancer is more effective through the approach of synthetic lethality. On the other hand, elevated FA function through gene copy increase or transcriptional regulation is a prevalent phenotype of a large cancer population and is widely associated with intrinsic or acquired resistance to ICL-inducing chemotherapy. For effective and novel treatments of cancers with FA alteration, incorporation of FA gene mutation status into tumor mutation screening and development of FA specific inhibitors are in demand.

## Data Availability

All data generated or analyzed during this study are included in this published article.

## Abbreviations

FA: Fanconi anemia
BER: Base excision repair
NER: Nucleotide excision repair
CIN: Chromosomal instability
ICL: Interstrand crosslink
NHEJ: Non-homologous end joining
MMEJ: Microhomology mediated end joining
DDR: DNA damage response
SSA: Single-stranded annealing

## References

[1] Sancar A, Lindsey-Boltz LA, Unsal-Kacmaz K, Linn S (2004). Molecular mechanisms of mammalian DNA repair and the DNA damage checkpoints. Annu Rev Biochem.

[2] Yoshikiyo K, Kratz K, Hirota K, Nishihara K, Takata M, Kurumizaka H, Horimoto S, Takeda S, Jiricny J (2010). KIAA1018/FAN1 nuclease protects cells against genomic instability induced by interstrand cross-linking agents. Proc Natl Acad Sci USA.

[3] Tubbs A, Nussenzweig A (2017). Endogenous DNA damage as a source of genomic instability in cancer. Cell.

[4] Deng CX (2006). BRCA1: cell cycle checkpoint, genetic instability, DNA damage response and cancer evolution. Nucleic Acids Res.

[5] Nagel ZD, Kitange GJ, Gupta SK, Joughin BA, Chaim IA, Mazzucato P, Lauffenburger DA, Sarkaria JN, Samson LD (2017). DNA repair capacity in multiple pathways predicts chemoresistance in glioblastoma multiforme. Can Res.

[6] Kim H, D'Andrea AD (2012). Regulation of DNA cross-link repair by the Fanconi anemia/BRCA pathway. Genes Dev.

[7] Niraj J, Farkkila A, D'Andrea AD (2019). The Fanconi anemia pathway in cancer. Annu Rev Cancer Biol.

[8] Rodriguez A, D'Andrea A (2017). Fanconi anemia pathway. Curr Biol.

[9] Ridpath JR, Nakamura A, Tano K, Luke AM, Sonoda E, Arakawa H, Buerstedde JM, Gillespie DA, Sale JE, Yamazoe M (2007). Cells deficient in the FANC/BRCA pathway are hypersensitive to plasma levels of formaldehyde. Can Res.

[10] Stone MP, Cho YJ, Huang H, Kim HY, Kozekov ID, Kozekova A, Wang H, Minko IG, Lloyd RS, Harris TM (2008). Interstrand DNA cross-links induced by alpha, beta-unsaturated aldehydes derived from lipid peroxidation and environmental sources. Acc Chem Res.

[11] Langevin F, Crossan GP, Rosado IV, Arends MJ, Patel KJ (2011). Fancd2 counteracts the toxic effects of naturally produced aldehydes in mice. Nature.

[12] Voulgaridou GP, Anestopoulos I, Franco R, Panayiotidis MI, Pappa A (2011). DNA damage induced by endogenous aldehydes: current state of knowledge. Mutat Res.

[13] O'Donovan A, Davies AA, Moggs JG, West SC, Wood RD (1994). XPG endonuclease makes the 3' incision in human DNA nucleotide excision repair. Nature.

[14] Clauson C, Scharer OD, Niedernhofer L (2013). Advances in understanding the complex mechanisms of DNA interstrand cross-link repair. Cold Spring Harb Perspect Biol.

[15] Sawyer SL, Tian L, Kahkonen M, Schwartzentruber J, Kircher M, Majewski J, Dyment DA, Innes AM, Boycott KM, Moreau LA (2015). Biallelic mutations in BRCA1 cause a new Fanconi anemia subtype. Cancer Discov.

[16] Dong H, Nebert DW, Bruford EA, Thompson DC, Joenje H, Vasiliou V (2015). Update of the human and mouse Fanconi anemia genes. Hum Genomics.

[17] Bogliolo M, Surralles J (2015). Fanconi anemia: a model disease for studies on human genetics and advanced therapeutics. Curr Opin Genet Dev.

[18] Ceccaldi R, Sarangi P, D'Andrea AD (2016). The Fanconi anaemia pathway: new players and new functions. Nat Rev Mol Cell Biol.

[19] Bluteau D, Masliah-Planchon J, Clairmont C, Rousseau A, Ceccaldi R, Dubois d’Enghien C, Bluteau O, Cuccuini W, Gachet S, Peffault de Latour R (2016). Biallelic inactivation of REV7 is associated with Fanconi anemia. J Clin Invest..

[20] Kottemann MC, Smogorzewska A (2013). Fanconi anaemia and the repair of Watson and Crick DNA crosslinks. Nature.

[21] Knies K, Inano S, Ramirez MJ, Ishiai M, Surralles J, Takata M, Schindler D (2017). Biallelic mutations in the ubiquitin ligase RFWD3 cause Fanconi anemia. J Clin Invest.

[22] Newell AE, Akkari YM, Torimaru Y, Rosenthal A, Reifsteck CA, Cox B, Grompe M, Olson SB (2004). Interstrand crosslink-induced radials form between non-homologous chromosomes, but are absent in sex chromosomes. DNA Repair (Amst).

[23] Deans AJ, West SC (2011). DNA interstrand crosslink repair and cancer. Nat Rev Cancer.

[24] Zhang J, Dewar JM, Budzowska M, Motnenko A, Cohn MA, Walter JC (2015). DNA interstrand cross-link repair requires replication-fork convergence. Nat Struct Mol Biol.

[25] Amunugama R, Willcox S, Wu RA, Abdullah UB, El-Sagheer AH, Brown T, McHugh PJ, Griffith JD, Walter JC (2018). Replication fork reversal during DNA interstrand crosslink repair requires CMG unloading. Cell Rep.

[26] Wu RA, Semlow DR, Kamimae-Lanning AN, Kochenova OV, Chistol G, Hodskinson MR, Amunugama R, Sparks JL, Wang M, Deng L (2019). TRAIP is a master regulator of DNA interstrand crosslink repair. Nature.

[27] Fullbright G, Rycenga HB, Gruber JD, Long DT (2016). p97 Promotes a conserved mechanism of helicase unloading during DNA cross-link repair. Mol Cell Biol.

[28] Ciccia A, Ling C, Coulthard R, Yan Z, Xue Y, Meetei AR, el Laghmani H, Joenje H, McDonald N, de Winter JP (2007). Identification of FAAP24, a Fanconi anemia core complex protein that interacts with FANCM. Mol Cell.

[29] Huang Y, Leung JW, Lowery M, Matsushita N, Wang Y, Shen X, Huong D, Takata M, Chen J, Li L (2014). Modularized functions of the Fanconi anemia core complex. Cell reports.

[30] Shakeel S, Rajendra E, Alcon P, O’Reilly F, Chorev DS, Maslen S, Degliesposti G, Russo CJ, He S, Hill CH (2019). Structure of the Fanconi anaemia monoubiquitin ligase complex. Nature.

[31] Swuec P, Renault L, Borg A, Shah F, Murphy VJ, van Twest S, Snijders AP, Deans AJ, Costa A (2017). The FA core complex contains a homo-dimeric catalytic module for the symmetric mono-ubiquitination of FANCI-FANCD2. Cell Rep.

[32] van Twest S, Murphy VJ, Hodson C, Tan W, Swuec P, O'Rourke JJ, Heierhorst J, Crismani W, Deans AJ (2017). Mechanism of ubiquitination and deubiquitination in the Fanconi anemia pathway. Mol Cell.

[33] Huang Y, Leung JW, Lowery M, Matsushita N, Wang Y, Shen X, Huong D, Takata M, Chen J, Li L (2014). Modularized functions of the Fanconi anemia core complex. Cell Rep.

[34] Hodson C, Purkiss A, Miles JA, Walden H (2014). Structure of the human FANCL RING-Ube2T complex reveals determinants of cognate E3-E2 selection. Structure..

[35] Smogorzewska A, Matsuoka S, Vinciguerra P, McDonald ER, Hurov KE, Luo J, Ballif BA, Gygi SP, Hofmann K, D'Andrea AD (2007). Identification of the FANCI protein, a monoubiquitinated FANCD2 paralog required for DNA repair. Cell.

[36] Joo W, Xu G, Persky NS, Smogorzewska A, Rudge DG, Buzovetsky O, Elledge SJ, Pavletich NP (2011). Structure of the FANCI-FANCD2 complex: insights into the Fanconi anemia DNA repair pathway. Science.

[37] Boisvert RA, Howlett NG (2014). The Fanconi anemia ID2 complex: dueling saxes at the crossroads. Cell Cycle.

[38] Sims AE, Spiteri E, Sims RJ, Arita AG, Lach FP, Landers T, Wurm M, Freund M, Neveling K, Hanenberg H (2007). FANCI is a second monoubiquitinated member of the Fanconi anemia pathway. Nat Struct Mol Biol.

[39] Alcon P, Shakeel S, Chen ZA, Rappsilber J, Patel KJ, Passmore LA (2020). FANCD2-FANCI is a clamp stabilized on DNA by monoubiquitination of FANCD2 during DNA repair. Nat Struct Mol Boil..

[40] Ishiai M, Kitao H, Smogorzewska A, Tomida J, Kinomura A, Uchida E, Saberi A, Kinoshita E, Kinoshita-Kikuta E, Koike T (2008). FANCI phosphorylation functions as a molecular switch to turn on the Fanconi anemia pathway. Nat Struct Mol Biol.

[41] Sareen A, Chaudhury I, Adams N, Sobeck A (2012). Fanconi anemia proteins FANCD2 and FANCI exhibit different DNA damage responses during S-phase. Nucleic Acids Res.

[42] Lopez-Martinez D, Kupculak M, Yang D, Yoshikawa Y, Liang CC, Wu R, Gygi SP, Cohn MA (2019). Phosphorylation of FANCD2 inhibits the FANCD2/FANCI complex and suppresses the Fanconi anemia pathway in the absence of DNA damage. Cell Rep..

[43] Liang F, Miller AS, Longerich S, Tang C, Maranon D, Williamson EA, Hromas R, Wiese C, Kupfer GM, Sung P (2019). DNA requirement in FANCD2 deubiquitination by USP1-UAF1-RAD51AP1 in the Fanconi anemia DNA damage response. Nat Commun.

[44] Arkinson C, Chaugule VK, Toth R, Walden H (2018). Specificity for deubiquitination of monoubiquitinated FANCD2 is driven by the N-terminus of USP1. Life Sci Alliance.

[45] Cukras S, Lee E, Palumbo E, Benavidez P, Moldovan GL, Kee Y (2016). The USP1-UAF1 complex interacts with RAD51AP1 to promote homologous recombination repair. Cell Cycle.

[46] Paquin KL, Mamrak NE, Garzon JL, Cantres-Velez JA, Azzinaro PA, Vuono EA, Lima KE, Camberg JL, Howlett NG (2019). FANCD2 binding to H4K20me2 via a methyl-binding domain is essential for efficient DNA cross-link repair. Mol Cell Biol.

[47] Knipscheer P, Raschle M, Smogorzewska A, Enoiu M, Ho TV, Scharer OD, Elledge SJ, Walter JC (2009). The Fanconi anemia pathway promotes replication-dependent DNA interstrand cross-link repair. Science.

[48] Benitez A, Yuan F, Nakajima S, Wei L, Qian L, Myers R, Hu JJ, Lan L, Zhang Y (2014). Damage-dependent regulation of MUS81-EME1 by Fanconi anemia complementation group a protein. Nucleic Acids Res.

[49] Bhagwat N, Olsen AL, Wang AT, Hanada K, Stuckert P, Kanaar R, D'Andrea A, Niedernhofer LJ, McHugh PJ (2009). XPF-ERCC1 participates in the fanconi anemia pathway of cross-link repair. Mol Cell Biol.

[50] Hanada K, Budzowska M, Davies SL, van Drunen E, Onizawa H, Beverloo HB, Maas A, Essers J, Hickson ID, Kanaar R (2007). The structure-specific endonuclease Mus81 contributes to replication restart by generating double-strand DNA breaks. Nat Struct Mol Biol.

[51] Hanada K, Budzowska M, Modesti M, Maas A, Wyman C, Essers J, Kanaar R (2006). The structure-specific endonuclease Mus81-Eme1 promotes conversion of interstrand DNA crosslinks into double-strands breaks. Embo J.

[52] Kim Y, Spitz GS, Veturi U, Lach FP, Auerbach AD, Smogorzewska A (2013). Regulation of multiple DNA repair pathways by the Fanconi anemia protein SLX4. Blood.

[53] Sengerova B, Wang AT, McHugh PJ (2011). Orchestrating the nucleases involved in DNA interstrand cross-link (ICL) repair. Cell Cycle.

[54] Wang AT, Sengerova B, Cattell E, Inagawa T, Hartley JM, Kiakos K, Burgess-Brown NA, Swift LP, Enzlin JH, Schofield CJ (2011). Human SNM1A and XPF-ERCC1 collaborate to initiate DNA interstrand cross-link repair. Genes Dev.

[55] Castella M, Taniguchi T (2010). The role of FAN1 nuclease in the Fanconi anemia pathway. Cell Cycle.

[56] Liu T, Ghosal G, Yuan J, Chen J, Huang J (2010). FAN1 acts with FANCI-FANCD2 to promote DNA interstrand cross-link repair. Science.

[57] MacKay C, Declais AC, Lundin C, Agostinho A, Deans AJ, MacArtney TJ, Hofmann K, Gartner A, West SC, Helleday T (2010). Identification of KIAA1018/FAN1, a DNA repair nuclease recruited to DNA damage by monoubiquitinated FANCD2. Cell.

[58] Shereda RD, Machida Y, Machida YJ (2010). Human KIAA1018/FAN1 localizes to stalled replication forks via its ubiquitin-binding domain. Cell Cycle.

[59] Smogorzewska A, Desetty R, Saito TT, Schlabach M, Lach FP, Sowa ME, Clark AB, Kunkel TA, Harper JW, Colaiacovo MP (2010). A genetic screen identifies FAN1, a Fanconi anemia-associated nuclease necessary for DNA interstrand crosslink repair. Mol Cell.

[60] Bogliolo M, Schuster B, Stoepker C, Derkunt B, Su Y, Raams A, Trujillo JP, Minguillon J, Ramirez MJ, Pujol R (2013). Mutations in ERCC4, encoding the DNA-repair endonuclease XPF, cause Fanconi anemia. Am J Hum Genet.

[61] Yamamoto KN, Kobayashi S, Tsuda M, Kurumizaka H, Takata M, Kono K, Jiricny J, Takeda S, Hirota K (2011). Involvement of SLX4 in interstrand cross-link repair is regulated by the Fanconi anemia pathway. Proc Natl Acad Sci USA.

[62] Hodskinson MR, Silhan J, Crossan GP, Garaycoechea JI, Mukherjee S, Johnson CM, Scharer OD, Patel KJ (2014). Mouse SLX4 is a tumor suppressor that stimulates the activity of the nuclease XPF-ERCC1 in DNA crosslink repair. Mol Cell.

[63] Zhang H, Chen Z, Ye Y, Ye Z, Cao D, Xiong Y, Srivastava M, Feng X, Tang M, Wang C (2019). SLX4IP acts with SLX4 and XPF-ERCC1 to promote interstrand crosslink repair. Nucleic Acids Res.

[64] Budzowska M, Graham TG, Sobeck A, Waga S, Walter JC (2015). Regulation of the Rev1-pol zeta complex during bypass of a DNA interstrand cross-link. EMBO J.

[65] Roy U, Scharer OD (2016). Involvement of translesion synthesis DNA polymerases in DNA interstrand crosslink repair. DNA Repair.

[66] Benitez A, Liu W, Palovcak A, Wang G, Moon J, An K, Kim A, Zheng K, Zhang Y, Bai F (2018). FANCA promotes DNA double-strand break repair by catalyzing single-strand annealing and strand exchange. Mol Cell.

[67] Ceccaldi R, Rondinelli B, D'Andrea AD (2016). Repair pathway choices and consequences at the double-strand break. Trends Cell Biol.

[68] Blier PR, Griffith AJ, Craft J, Hardin JA (1993). Binding of Ku protein to DNA. Measurement of affinity for ends and demonstration of binding to nicks. J Biol Chem..

[69] Falzon M, Fewell JW, Kuff EL (1993). EBP-80, a transcription factor closely resembling the human autoantigen Ku, recognizes single- to double-strand transitions in DNA. J Biol Chem.

[70] Mimori T, Hardin JA (1986). Mechanism of interaction between Ku protein and DNA. J Biol Chem.

[71] Symington LS, Gautier J (2011). Double-strand break end resection and repair pathway choice. Annu Rev Genet.

[72] Ochs F, Somyajit K, Altmeyer M, Rask MB, Lukas J, Lukas C (2016). 53BP1 fosters fidelity of homology-directed DNA repair. Nat Struct Mol Biol.

[73] Ceccaldi R, Liu JC, Amunugama R, Hajdu I, Primack B, Petalcorin MI, O'Connor KW, Konstantinopoulos PA, Elledge SJ, Boulton SJ (2015). Homologous-recombination-deficient tumours are dependent on Poltheta-mediated repair. Nature.

[74] Bhargava R, Onyango DO, Stark JM (2016). Regulation of single-strand annealing and its role in genome maintenance. Trends Genet.

[75] Palovcak A, Liu W, Yuan F, Zhang Y (2017). Maintenance of genome stability by Fanconi anemia proteins. Cell Biosci.

[76] Murina O, von Aesch C, Karakus U, Ferretti LP, Bolck HA, Hanggi K, Sartori AA (2014). FANCD2 and CtIP cooperate to repair DNA interstrand crosslinks. Cell Rep.

[77] Ohashi A, Zdzienicka MZ, Chen J, Couch FJ (2005). Fanconi anemia complementation group D2 (FANCD2) functions independently of BRCA2- and RAD51-associated homologous recombination in response to DNA damage. J Biol Chem.

[78] Nakanishi K, Yang YG, Pierce AJ, Taniguchi T, Digweed M, D'Andrea AD, Wang ZQ, Jasin M (2005). Human Fanconi anemia monoubiquitination pathway promotes homologous DNA repair. Proc Natl Acad Sci USA.

[79] Adamo A, Collis SJ, Adelman CA, Silva N, Horejsi Z, Ward JD, Martinez-Perez E, Boulton SJ, La Volpe A (2010). Preventing nonhomologous end joining suppresses DNA repair defects of Fanconi anemia. Mol Cell.

[80] Pace P, Mosedale G, Hodskinson MR, Rosado IV, Sivasubramaniam M, Patel KJ (2010). Ku70 corrupts DNA repair in the absence of the Fanconi anemia pathway. Science.

[81] Yuan F, Qian L, Zhao X, Liu JY, Song L, D'Urso G, Jain C, Zhang Y (2012). Fanconi anemia complementation group A (FANCA) protein has intrinsic affinity for nucleic acids with preference for single-stranded forms. J Biol Chem.

[82] Palovcak A, Liu W, Yuan F, Zhang Y (2018). Stitching up broken DNA ends by FANCA. Mol Cell Oncol.

[83] Steinberg-Shemer O, Goldberg TA, Yacobovich J, Levin C, Koren A, Revel-Vilk S, Ben-Ami T, Kuperman AA, Shkalim Zemer V, Toren A (2019). Characterization and genotype-phenotype correlation of patients with Fanconi anemia in a multi-ethnic population. Haematologica.

[84] Kennedy RD, D'Andrea AD (2005). The Fanconi Anemia/BRCA pathway: new faces in the crowd. Genes Dev.

[85] Quentin S, Cuccuini W, Ceccaldi R, Nibourel O, Pondarre C, Pages MP, Vasquez N, Dubois d’Enghien C, Larghero J, Peffault de Latour R (2011). Myelodysplasia and leukemia of Fanconi anemia are associated with a specific pattern of genomic abnormalities that includes cryptic RUNX1/AML1 lesions. Blood.

[86] Bakker ST, de Winter JP, te Riele H (2013). Learning from a paradox: recent insights into Fanconi anaemia through studying mouse models. Dis Models Mech.

[87] Parmar K, D'Andrea A, Niedernhofer LJ (2009). Mouse models of Fanconi anemia. Mutat Res.

[88] Hanahan D, Weinberg RA (2011). Hallmarks of cancer: the next generation. Cell.

[89] Crossley MP, Bocek M, Cimprich KA (2019). R-loops as cellular regulators and genomic threats. Mol Cell.

[90] Skourti-Stathaki K, Proudfoot NJ (2014). A double-edged sword: R loops as threats to genome integrity and powerful regulators of gene expression. Genes Dev.

[91] Gan W, Guan Z, Liu J, Gui T, Shen K, Manley JL, Li X (2011). R-loop-mediated genomic instability is caused by impairment of replication fork progression. Genes Dev.

[92] Garcia-Rubio ML, Perez-Calero C, Barroso SI, Tumini E, Herrera-Moyano E, Rosado IV, Aguilera A (2015). The Fanconi anemia pathway protects genome integrity from R-loops. PLoS Genet.

[93] Hamperl S, Bocek MJ, Saldivar JC, Swigut T, Cimprich KA (2017). Transcription-replication conflict orientation modulates R-loop levels and activates distinct DNA damage responses. Cell.

[94] Schwab RA, Nieminuszczy J, Shah F, Langton J, Lopez Martinez D, Liang CC, Cohn MA, Gibbons RJ, Deans AJ, Niedzwiedz W (2015). The Fanconi anemia pathway maintains genome stability by coordinating replication and transcription. Mol Cell.

[95] Liang Z, Liang F, Teng Y, Chen X, Liu J, Longerich S, Rao T, Green AM, Collins NB, Xiong Y (2019). Binding of FANCI-FANCD2 complex to RNA and R-loops stimulates robust FANCD2 monoubiquitination. Cell Rep.

[96] Okamoto Y, Hejna J, Takata M (2019). Regulation of R-loops and genome instability in Fanconi anemia. J Biochem.

[97] Okamoto Y, Abe M, Itaya A, Tomida J, Ishiai M, Takaori-Kondo A, Taoka M, Isobe T, Takata M (2019). FANCD2 protects genome stability by recruiting RNA processing enzymes to resolve R-loops during mild replication stress. FEBS J.

[98] Hoskins EE, Gunawardena RW, Habash KB, Wise-Draper TM, Jansen M, Knudsen ES, Wells SI (2008). Coordinate regulation of Fanconi anemia gene expression occurs through the Rb/E2F pathway. Oncogene.

[99] Taniguchi T, Tischkowitz M, Ameziane N, Hodgson SV, Mathew CG, Joenje H, Mok SC, D'Andrea AD (2003). Disruption of the Fanconi anemia-BRCA pathway in cisplatin-sensitive ovarian tumors. Nat Med.

[100] Tremblay CS, Huang FF, Habi O, Huard CC, Godin C, Levesque G, Carreau M (2008). HES1 is a novel interactor of the Fanconi anemia core complex. Blood.

[101] Tremblay CS, Huard CC, Huang FF, Habi O, Bourdages V, Levesque G, Carreau M (2009). The fanconi anemia core complex acts as a transcriptional co-regulator in hairy enhancer of split 1 signaling. J Biol Chem.

[102] Huard CC, Tremblay CS, Magron A, Levesque G, Carreau M (2014). The Fanconi anemia pathway has a dual function in Dickkopf-1 transcriptional repression. Proc Natl Acad Sci USA.

[103] Nguyen B, Gao L, Almiman A, Tang S, Dotts K, Villalona-Calero MA, Duan W (2019). Abstract 2568: investigation of Fanconi anemia pathway downstream genes. Cancer Res..

[104] Jung Y, Lippard SJ (2007). Direct cellular responses to platinum-induced DNA damage. Chem Rev.

[105] Chirnomas D, Taniguchi T, de la Vega M, Vaidya AP, Vasserman M, Hartman AR, Kennedy R, Foster R, Mahoney J, Seiden MV (2006). Chemosensitization to cisplatin by inhibitors of the Fanconi anemia/BRCA pathway. Mol Cancer Ther.

[106] Helleday T, Petermann E, Lundin C, Hodgson B, Sharma RA (2008). DNA repair pathways as targets for cancer therapy. Nat Rev Cancer.

[107] Chen P, Li J, Chen YC, Qian H, Chen YJ, Su JY, Wu M, Lan T (2016). The functional status of DNA repair pathways determines the sensitization effect to cisplatin in non-small cell lung cancer cells. Cell Oncol.

[108] Chen Q, Van der Sluis PC, Boulware D, Hazlehurst LA, Dalton WS (2005). The FA/BRCA pathway is involved in melphalan-induced DNA interstrand cross-link repair and accounts for melphalan resistance in multiple myeloma cells. Blood.

[109] van der Heijden MS, Brody JR, Dezentje DA, Gallmeier E, Cunningham SC, Swartz MJ, DeMarzo AM, Offerhaus GJ, Isacoff WH, Hruban RH (2005). In vivo therapeutic responses contingent on Fanconi anemia/BRCA2 status of the tumor. Clin Cancer Res.

[110] Chen CC, Taniguchi T, D'Andrea A (2007). The Fanconi anemia (FA) pathway confers glioma resistance to DNA alkylating agents. J Mol Med.

[111] Bravo-Navas S, Yanez L, Romon I, Pipaon C (2019). Elevated FANCA expression determines a worse prognosis in chronic lymphocytic leukemia and interferes with p53 function. FASEB J.

[112] Zhang W, Zhang Y, Yang Z, Liu X, Yang P, Wang J, Hu K, He X, Zhang X, Jing H (2019). High expression of UBE2T predicts poor prognosis and survival in multiple myeloma. Cancer Gene Ther.

[113] Dietlein F, Thelen L, Reinhardt HC (2014). Cancer-specific defects in DNA repair pathways as targets for personalized therapeutic approaches. Trends Genet.

[114] Negrini S, Gorgoulis VG, Halazonetis TD (2010). Genomic instability–an evolving hallmark of cancer. Nat Rev Mol Cell Biol.

[115] Fong PC, Boss DS, Yap TA, Tutt A, Wu P, Mergui-Roelvink M, Mortimer P, Swaisland H, Lau A, O'Connor MJ (2009). Inhibition of poly(ADP-ribose) polymerase in tumors from BRCA mutation carriers. N Engl J Med.

[116] Patel AG, Sarkaria JN, Kaufmann SH (2011). Nonhomologous end joining drives poly(ADP-ribose) polymerase (PARP) inhibitor lethality in homologous recombination-deficient cells. Proc Natl Acad Sci USA.

[117] Mendes-Pereira AM, Martin SA, Brough R, McCarthy A, Taylor JR, Kim JS, Waldman T, Lord CJ, Ashworth A (2009). Synthetic lethal targeting of PTEN mutant cells with PARP inhibitors. EMBO Mol Med.

[118] Bian X, Gao J, Luo F, Rui C, Zheng T, Wang D, Wang Y, Roberts TM, Liu P, Zhao JJ (2018). PTEN deficiency sensitizes endometrioid endometrial cancer to compound PARP-PI3K inhibition but not PARP inhibition as monotherapy. Oncogene.

[119] Fraser M, Zhao H, Luoto KR, Lundin C, Coackley C, Chan N, Joshua AM, Bismar TA, Evans A, Helleday T (2012). PTEN deletion in prostate cancer cells does not associate with loss of RAD51 function: implications for radiotherapy and chemotherapy. Clin Cancer Res.

[120] Ye H, Zhang X, Chen Y, Liu Q, Wei J (2016). Ranking novel cancer driving synthetic lethal gene pairs using TCGA data. Oncotarget.

[121] Kennedy RD, Chen CC, Stuckert P, Archila EM, De la Vega MA, Moreau LA, Shimamura A, D'Andrea AD (2007). Fanconi anemia pathway-deficient tumor cells are hypersensitive to inhibition of ataxia telangiectasia mutated. J Clin Invest.

[122] Rosado IV, Langevin F, Crossan GP, Takata M, Patel KJ (2011). Formaldehyde catabolism is essential in cells deficient for the Fanconi anemia DNA-repair pathway. Nat Struct Mol Biol.

[123] Tsherniak A, Vazquez F, Montgomery PG, Weir BA, Kryukov G, Cowley GS, Gill S, Harrington WF, Pantel S, Krill-Burger JM (2017). Defining a cancer dependency map. Cell.

[124] Jacquemont C, Taniguchi T (2007). Proteasome function is required for DNA damage response and fanconi anemia pathway activation. Can Res.

[125] Duan W, Gao L, Zhao W, Leon M, Sadee W, Webb A, Resnick K, Wu X, Ramaswamy B, Cohn DE (2013). Assessment of FANCD2 nuclear foci formation in paraffin-embedded tumors: a potential patient-enrichment strategy for treatment with DNA interstrand crosslinking agents. Transl Res.

[126] Landais I, Hiddingh S, McCarroll M, Yang C, Sun A, Turker MS, Snyder JP, Hoatlin ME (2009). Monoketone analogs of curcumin, a new class of Fanconi anemia pathway inhibitors. Mol Cancer.

[127] Oda T, Hayano T, Miyaso H, Takahashi N, Yamashita T (2007). Hsp90 regulates the Fanconi anemia DNA damage response pathway. Blood.

[128] Liu W, Wang G, Palovcak A, Li Y, Hao S, Liu ZJ, Landgraf R, Yuan F, Zhang Y (2019). Impeding the single-strand annealing pathway of DNA double-strand break repair by withaferin A-mediated FANCA degradation. DNA Repair (Amst).

[129] Sijbers AM, van der Spek PJ, Odijk H, van den Berg J, van Duin M, Westerveld A, Jaspers NG, Bootsma D, Hoeijmakers JH (1996). Mutational analysis of the human nucleotide excision repair gene ERCC1. Nucleic Acids Res.

[130] de Laat WL, Sijbers AM, Odijk H, Jaspers NG, Hoeijmakers JH (1998). Mapping of interaction domains between human repair proteins ERCC1 and XPF. Nucleic Acids Res.

[131] Cornwell MJ, Thomson GJ, Coates J, Belotserkovskaya R, Waddell ID, Jackson SP, Galanty Y (2019). Small-molecule inhibition of UBE2T/FANCL-mediated ubiquitylation in the Fanconi anemia Pathway. ACS Chem Biol.

[132] Jeong E, Lee SG, Kim HS, Yang J, Shin J, Kim Y, Kim J, Scharer OD, Kim Y, Yeo JE (2020). Structural basis of the fanconi anemia-associated mutations within the FANCA and FANCG complex. Nucleic Acids Res.

[133] Kowal P, Gurtan AM, Stuckert P, D'Andrea AD, Ellenberger T (2007). Structural determinants of human FANCF protein that function in the assembly of a DNA damage signaling complex. J Biol Chem.

[134] Arai MA, Uemura K, Hamahiga N, Ishikawa N, Koyano T, Kowithayakorn T, Kaddar T, Carreau M, Ishibashi M (2015). Naturally occurring FANCF-Hes1 complex inhibitors from Wrightia religiosa. Medchemcomm.

[135] Hodson C, Cole AR, Lewis LP, Miles JA, Purkiss A, Walden H (2011). Structural analysis of human FANCL, the E3 ligase in the Fanconi anemia pathway. J Biol Chem.

[136] Coulthard R, Deans AJ, Swuec P, Bowles M, Costa A, West SC, McDonald NQ (2013). Architecture and DNA recognition elements of the Fanconi anemia FANCM-FAAP24 complex. Structure.

[137] Hoadley KA, Xue Y, Ling C, Takata M, Wang W, Keck JL (2012). Defining the molecular interface that connects the Fanconi anemia protein FANCM to the Bloom syndrome dissolvasome. Proc Natl Acad Sci USA.

[138] Tao Y, Jin C, Li X, Qi S, Chu L, Niu L, Yao X, Teng M (2012). The structure of the FANCM-MHF complex reveals physical features for functional assembly. Nat Commun.

[139] Yang H, Zhang T, Tao Y, Wang F, Tong L, Ding J (2013). Structural insights into the functions of the FANCM-FAAP24 complex in DNA repair. Nucleic Acids Res.

[140] Voter AF, Manthei KA, Keck JL (2016). A High-Throughput screening strategy to identify protein-protein interaction inhibitors that block the Fanconi anemia DNA repair pathway. J Biomol Screen.

[141] Lu R, O’Rourke JJ, Sobinoff AP, Allen JAM, Nelson CB, Tomlinson CG, Lee M (2019). The FANCM-BLM-TOP3A-RMI complex suppresses alternative lengthening of telomeres (ALT). Nat Commun..

[142] Sheng Y, Hong JH, Doherty R, Srikumar T, Shloush J, Avvakumov GV, Walker JR, Xue S, Neculai D, Wan JW (2012). A human ubiquitin conjugating enzyme (E2)-HECT E3 ligase structure-function screen. Mol Cell Proteomics.

[143] Morreale FE, Bortoluzzi A, Chaugule VK, Arkinson C, Walden H, Ciulli A (2017). Allosteric targeting of the Fanconi anemia ubiquitin-conjugating enzyme Ube2T by fragment screening. J Med Chem..

[144] Morreale FE, Testa A (2017). Mind the metal: A fragment library-derived zinc impurity binds the E2 ubiquitin-conjugating enzyme Ube2T and induces structural rearrangements. J Med Chem..

[145] Park WH, Margossian S, Horwitz AA, Simons AM, D'Andrea AD, Parvin JD (2005). Direct DNA binding activity of the Fanconi anemia D2 protein. J Biol Chem.

[146] Yuan F, El Hokayem J, Zhou W, Zhang Y (2009). FANCI protein binds to DNA and interacts with FANCD2 to recognize branched structures. J Biol Chem.

[147] Longerich S, San Filippo J, Liu D, Sung P (2009). FANCI binds branched DNA and is monoubiquitinated by UBE2T-FANCL. J Biol Chem.

[148] Wan B, Yin J, Horvath K, Sarkar J, Chen Y, Wu J, Wan K, Lu J, Gu P, Yu EY (2013). SLX4 assembles a telomere maintenance toolkit by bridging multiple endonucleases with telomeres. Cell reports.

[149] Pellegrini L, Yu DS, Lo T, Anand S, Lee M, Blundell TL, Venkitaraman AR (2002). Insights into DNA recombination from the structure of a RAD51-BRCA2 complex. Nature.

[150] Oliver AW, Swift S, Lord CJ, Ashworth A, Pearl LH (2009). Structural basis for recruitment of BRCA2 by PALB2. EMBO Rep.

[151] Rytelewski M, Tong JG, Buensuceso A, Leong HS, Maleki Vareki S, Figueredo R, Di Cresce C, Wu SY, Herbrich SM, Baggerly KA (2014). BRCA2 inhibition enhances cisplatin-mediated alterations in tumor cell proliferation, metabolism, and metastasis. Mol Oncol.

[152] Clapperton JA, Manke IA, Lowery DM, Ho T, Haire LF, Yaffe MB, Smerdon SJ (2004). Structure and mechanism of BRCA1 BRCT domain recognition of phosphorylated BACH1 with implications for cancer. Nat Struct Mol Biol.

[153] Shiozaki EN, Gu L, Yan N, Shi Y (2004). Structure of the BRCT repeats of BRCA1 bound to a BACH1 phosphopeptide: implications for signaling. Mol Cell.

[154] Leung CC, Gong Z, Chen J, Glover JN (2011). Molecular basis of BACH1/FANCJ recognition by TopBP1 in DNA replication checkpoint control. J Biol Chem.

[155] Aihara H, Ito Y, Kurumizaka H, Yokoyama S, Shibata T (1999). The N-terminal domain of the human Rad51 protein binds DNA: structure and a DNA binding surface as revealed by NMR. J Mol Biol.

[156] Xu J, Zhao L, Xu Y, Zhao W, Sung P, Wang HW (2017). Cryo-EM structures of human RAD51 recombinase filaments during catalysis of DNA-strand exchange. Nat Struct Mol Biol.

[157] Short JM, Liu Y, Chen S, Soni N, Madhusudhan MS, Shivji MK, Venkitaraman AR (2016). High-resolution structure of the presynaptic RAD51 filament on single-stranded DNA by electron cryo-microscopy. Nucleic Acids Res.

[158] Brouwer I, Moschetti T, Candelli A, Garcin EB, Modesti M, Pellegrini L (2018). Two distinct conformational states define the interaction of human RAD51-ATP with single-stranded DNA. EMBO J..

[159] Budke B, Kalin JH, Pawlowski M, Zelivianskaia AS, Wu M, Kozikowski AP, Connell PP (2013). An optimized RAD51 inhibitor that disrupts homologous recombination without requiring Michael acceptor reactivity. J Med Chem.

[160] Huang F, Motlekar NA, Burgwin CM, Napper AD, Diamond SL, Mazin AV (2011). Identification of specific inhibitors of human RAD51 recombinase using high-throughput screening. ACS Chem Biol.

[161] Budke B, Lv W, Kozikowski AP, Connell PP (2016). Recent developments using small molecules to target RAD51: How to best modulate RAD51 for anticancer therapy?. Chem Med Chem.

[162] Brzovic PS, Rajagopal P, Hoyt DW, King MC, Klevit RE (2001). Structure of a BRCA1-BARD1 heterodimeric RING-RING complex. Nat Struct Biol.

[163] Williams RS, Green R, Glover JN (2001). Crystal structure of the BRCT repeat region from the breast cancer-associated protein BRCA1. Nat Struct Biol.

[164] Williams RS, Glover JN (2003). Structural consequences of a cancer-causing BRCA1-BRCT missense mutation. J Biol Chem.

[165] Gaiser OJ, Ball LJ, Schmieder P, Leitner D, Strauss H, Wahl M, Kuhne R, Oschkinat H, Heinemann U (2004). Solution structure, backbone dynamics, and association behavior of the C-terminal BRCT domain from the breast cancer-associated protein BRCA1. Biochemistry.

[166] Williams RS, Lee MS, Hau DD, Glover JN (2004). Structural basis of phosphopeptide recognition by the BRCT domain of BRCA1. Nat Struct Mol Biol.

[167] Varma AK, Brown RS, Birrane G, Ladias JA (2005). Structural basis for cell cycle checkpoint control by the BRCA1-CtIP complex. Biochemistry.

[168] Shen Y, Tong L (2008). Structural evidence for direct interactions between the BRCT domains of human BRCA1 and a phospho-peptide from human ACC1. Biochemistry.

[169] Campbell SJ, Edwards RA, Glover JN (2010). Comparison of the structures and peptide binding specificities of the BRCT domains of MDC1 and BRCA1. Structure..

[170] Coquelle N, Green R, Glover JN (2011). Impact of BRCA1 BRCT domain missense substitutions on phosphopeptide recognition. Biochemistry.

[171] Liu X, Ladias JA (2013). Structural basis for the BRCA1 BRCT interaction with the proteins ATRIP and BAAT1. Biochemistry.

[172] White ER, Sun L, Ma Z, Beckta JM, Danzig BA, Hacker DE, Huie M, Williams DC, Edwards RA, Valerie K (2015). Peptide library approach to uncover phosphomimetic inhibitors of the BRCA1 C-terminal domain. ACS Chem Biol.

[173] Wu Q, Paul A, Su D, Mehmood S, Foo TK, Ochi T, Bunting EL, Xia B, Robinson CV, Wang B (2016). Structure of BRCA1-BRCT/Abraxas complex reveals phosphorylation-dependent BRCT dimerization at DNA damage sites. Mol Cell.

[174] Hunkeler M, Hagmann A, Stuttfeld E, Chami M, Guri Y, Stahlberg H, Maier T (2018). Structural basis for regulation of human acetyl-CoA carboxylase. Nature.

[175] Hara K, Hashimoto H, Murakumo Y, Kobayashi S, Kogame T, Unzai S, Akashi S, Takeda S, Shimizu T, Sato M (2010). Crystal structure of human REV7 in complex with a human REV3 fragment and structural implication of the interaction between DNA polymerase zeta and REV1. J Biol Chem.

[176] Kikuchi S, Hara K, Shimizu T, Sato M, Hashimoto H (2012). Structural basis of recruitment of DNA polymerase zeta by interaction between REV1 and REV7 proteins. J Biol Chem.

[177] Xie W, Yang X, Xu M, Jiang T (2012). Structural insights into the assembly of human translesion polymerase complexes. Protein Cell.

[178] Hara K, Taharazako S, Ikeda M, Fujita H, Mikami Y, Kikuchi S, Hishiki A, Yokoyama H, Ishikawa Y, Kanno SI (2017). Dynamic feature of mitotic arrest deficient 2-like protein 2 (MAD2L2) and structural basis for its interaction with chromosome alignment-maintaining phosphoprotein (CAMP). J Biol Chem..

[179] Rizzo AA, Vassel FM, Chatterjee N, D'Souza S, Li Y, Hao B (2018). Rev7 dimerization is important for assembly and function of the Rev1/Polzeta translesion synthesis complex. Proc Natl Acad Sci USA.

[180] Actis ML, Ambaye ND, Evison BJ, Shao Y, Vanarotti M, Inoue A, McDonald ET, Kikuchi S, Heath R, Hara K (2016). Identification of the first small-molecule inhibitor of the REV7 DNA repair protein interaction. Bioorg Med Chem.

[181] Tripsianes K, Folkers G, Ab E, Das D, Odijk H, Jaspers NG, Hoeijmakers JH, Kaptein R, Boelens R (2005). The structure of the human ERCC1/XPF interaction domains reveals a complementary role for the two proteins in nucleotide excision repair. Structure.

[182] Tsodikov OV, Enzlin JH, Scharer OD, Ellenberger T (2005). Crystal structure and DNA binding functions of ERCC1, a subunit of the DNA structure-specific endonuclease XPF-ERCC1. Proc Natl Acad Sci USA.

[183] Das D, Tripsianes K, Jaspers NG, Hoeijmakers JH, Kaptein R, Boelens R, Folkers GE (2008). The HhH domain of the human DNA repair protein XPF forms stable homodimers. Proteins.

[184] Das D, Folkers GE, van Dijk M, Jaspers NG, Hoeijmakers JH, Kaptein R, Boelens R (2012). The structure of the XPF-ssDNA complex underscores the distinct roles of the XPF and ERCC1 helix-hairpin-helix domains in ss/ds DNA recognition. Structure..

[185] Faridounnia M, Wienk H, Kovacic L, Folkers GE, Jaspers NG, Kaptein R, Hoeijmakers JH, Boelens R (2015). The Cerebro-oculo-facio-skeletal syndrome point mutation F231L in the ERCC1 DNA repair protein causes dissociation of the ERCC1-XPF complex. J biol Chem.

